# A randomized controlled pilot study assessing feasibility and safety of a wilderness program for childhood, adolescent, and young adult cancer survivors: the WAYA study

**DOI:** 10.1186/s12889-023-16408-x

**Published:** 2023-08-08

**Authors:** Miek C. Jong, Heléne Dahlqvist, E. Anne Lown, Winnie Schats, Lars Beckman, Mats Jong

**Affiliations:** 1 Department of Health Sciences, Mid Sweden University, Holmgatan 10, 851 70 Sundsvall, Sweden; 2 The Arctic University of Norway, National Research Center in Complementary and Alternative Medicine (NAFKAM), Department of Community Medicine, Faculty of Health Sciences, UiT, Hansine Hansens Veg 18, 9019 Tromsø, Norway; 3 Department of Social & Behavioral Sciences, University of California San Francisco, 490 Illinois Street, SBS, Box 0612, San Francisco, CA 94143 USA; 4 Scientific Information Service, Netherlands Cancer Institute, Plesmanlaan 121, 1066 CX Amsterdam, The Netherlands; 5 Department of Oncology, Sundsvall Hospital, 85186 Sundsvall, Sweden

**Keywords:** Childhood cancer, Health promotion, Nature, Nature-connectedness, Oncology, Quality of life

## Abstract

**Background:**

The majority of childhood, Adolescent and Young Adult (AYA) cancer survivors suffers from long-lasting health issues following cancer treatment. It is therefore critical to explore effective health promotion strategies to address their needs. Exposure to nature is a promising approach to support the needs of young cancer survivors. This study investigated whether it is feasible to conduct a randomized controlled trial (RCT) of a wilderness program for childhood and AYA cancer survivors.

**Methods:**

Eligible participants were aged 16–39 years, had a cancer diagnosis, and met minimal criteria. Seventy-one individuals expressed interest and 59 were randomized to either a wilderness or a holiday program. The wilderness program involved an 8-day expedition including backpacking, sea kayaking, gorge climbing, camping, bush-craft skills, and mindfulness-practices. It was followed by a 4-day basecamp after 3 months. The comparison was an 8-day holiday program at a Spa-hotel followed by a 4-day holiday program at the same hotel after 3 months. Primary outcome was study feasibility and safety.

**Results:**

Ultimately, 19 AYAs participated in the wilderness and 23 in the holiday program. All completed the study at one-year follow-up. Participants were mostly female (70%) and represented diverse cancers. Clinical characteristics were similar between study arms excepting greater age at cancer diagnosis in the wilderness program (age 19.1 vs. 12.5; *p* = 0.024). Program adherence and data completeness was high (> 90%) in both arms. Adverse Effects (AEs) in the wilderness vs. the holiday program were similar (Relative Risk: 1.0, 95% Confidence Interval 0.8–1.3). The most frequent AE was tiredness, all were mild to moderate in severity, and serious AEs were not reported. Nature connectedness significantly increased over time in the wilderness program participants, but not in the holiday program (*p* < 0.001). No differences were found between the two study arms regarding quality of life, self-esteem, or self-efficacy.

**Conclusion:**

It is feasible to conduct a RCT and a supervised wilderness adventure is equally safe for childhood and AYA cancer survivors as a holiday program. This pilot study lays the foundation for a larger RCT to investigate the effectiveness of wilderness programs on the health of young cancer survivors.

**Trial registration date and number:**

18/02/2021, NCT04761042 (clinicaltrials.gov).

**Supplementary Information:**

The online version contains supplementary material available at 10.1186/s12889-023-16408-x.

## Background

In the past decades, five-year overall survival rates for childhood and Adolescent and Young Adults (AYA) cancer survivors have increased to 86% [1–3]. This steadily increasing cancer survivor population is however at higher risk of developing secondary malignancies, and they have almost double the risk of dying from a new primary cancer compared to the general population [4]. AYA cancer survivors have a greater risk for other late effects from their cancer or cancer treatment, such as cardiovascular disorders, endocrinopathies, gonadal dysfunction, neurological disorders, diabetes, and osteoporosis [3, 5, 6]. Health care utilization and medical expenditures are high in this group [7]. Furthermore, psychological distress is more common in AYA cancer survivors compared to their siblings or the general population [3, 8–10]. The higher prevalence of psychological distress in AYA cancer survivors is influenced by multiple complex issues from a cancer diagnosis, treatment and stage in life, where issues arise related to fertility, education, early-career goals, social interactions, family functioning, and financial status [5, 11, 12]. Survivors of childhood cancer reaching adulthood face similar late effects and challenges as those described above for AYA cancer survivors [13]. A substantial subgroup of childhood cancer survivors experience general psychological distress [14], and nearly 16% have posttraumatic stress disorder in young adulthood [15]. Furthermore, long-term childhood cancer survivors are more likely to be frail (reduced physiologic reserve often seen in the elderly) with 7.9% of young adult survivors meeting that criterion [16, 17]. The long-term childhood cancer survivor population has reached 500,000 in the United States [18, 19]. The yearly incidence rate for cancer in the age group 0–19 years is 18.5 per 100, 000 in Sweden and 83.0 per 100, 000 for those aged between 20–39 years [20].

It is of utmost importance to provide childhood and AYA cancers survivors with effective interventions and rehabilitation- or health promotion programs during their survivorship to support them in meeting physical, psychological, and social challenges. A systematic review and meta-analysis of psychosocial interventions for AYA cancer survivors demonstrated that technology-based as well as psycho-educational, multi-component and physical exercise interventions have very small and non-significant effects in this target group [21]. Most of these interventions were not specifically developed for AYAs, studies were small, and did not examine psychosocial parameters as primary outcomes [21]. Similar inconclusive evidence was reported in another systematic review on existing health promotion and psychological interventions for AYA cancer survivors mostly due to weak methodology [22]. (Online) peer-support groups have been reported as helpful for coping with psychological distress by AYA cancer survivors, but its effectiveness on health-related outcomes has not yet been demonstrated [23]. Until now, technology-assisted interventions [24, 25] and physical activity-based interventions [26] seem to be most promising in the support of childhood and AYA cancer survivors. Two recent meta-analyses showed that technology-assisted psychosocial interventions, such as e-health and distraction interventions, are effective in improving psychosocial and emotional health [24, 25]. However, favourable evidence was found primarily for childhood cancer survivors with limited support for AYA cancer survivors [25]. A previous systematic review and meta-analysis on physical activity demonstrated a pooled overall effect on quality of life of AYA cancer survivors in favour of physical exercise interventions, but with marginal significance [27].

Given the multitude of chronic health issues among childhood and AYA cancer survivors and given that there are few interventions that have the potential to impact on multiple factors, we developed and piloted a multi-component health promotion intervention: The Wilderness program for childhood, Adolescent and Young Adults (WAYA) program [28]. Adventure- or wilderness therapy could be a promising intervention to better support the needs of childhood and AYA cancer survivors [29, 30]. Although there is no universally accepted definition for these type of interventions, adventure therapy is commonly regarded as the umbrella term under which a large variety of other outdoor programs appear [31].

Exposure to nature has shown to positively affect our health and well-being [32, 33], involving a range of potential mechanisms [34]. Time in nature has shown to increase physical activity [35], to enhance the functional health benefits from physical exercise [36], and to increase social contact and community feeling [37, 38]. In addition, natural environments promote restorative mental functioning [39] and a walk in nature has been shown to reduce stress-related brain activity [40]. A recently published scoping review reported that wilderness programs may increase social involvement, self-esteem, self-confidence, self-efficacy, social support, and physical activity among childhood and AYA cancer survivors [41]. However, the following gaps on the topic were identified: 1) The absence of randomized controlled trials (RCTs) to investigate the effectiveness of wilderness programs for AYA cancer survivors; 2) Lack of studies on long-term health-related effects of these programs; 3) Little to no information on the safety of wilderness programs in this specific target group [41]. It was concluded that pragmatic RCTs are necessary to further investigate the effectiveness and safety of wilderness programs in childhood and AYA cancer survivors [41]. Performing randomized designs on wilderness programs is challenging. A previous Norwegian RCT on a wilderness intervention for adolescents with mental health problems had to change its design when controls, who received care as usual, dropped out preventing implementation of the randomized design [42]. Therefore, the present study was initiated to examine whether it would be feasible to perform a randomized study design on a wilderness intervention among childhood and AYA cancer survivors. The primary aim of this study was thus to investigate the feasibility and safety of performing a RCT on a wilderness program for childhood and AYA cancer survivors. The secondary aim was to explore the impact of this wilderness program on participant health and well-being.

## Methods

### Study design and setting

**Fig. 1:**
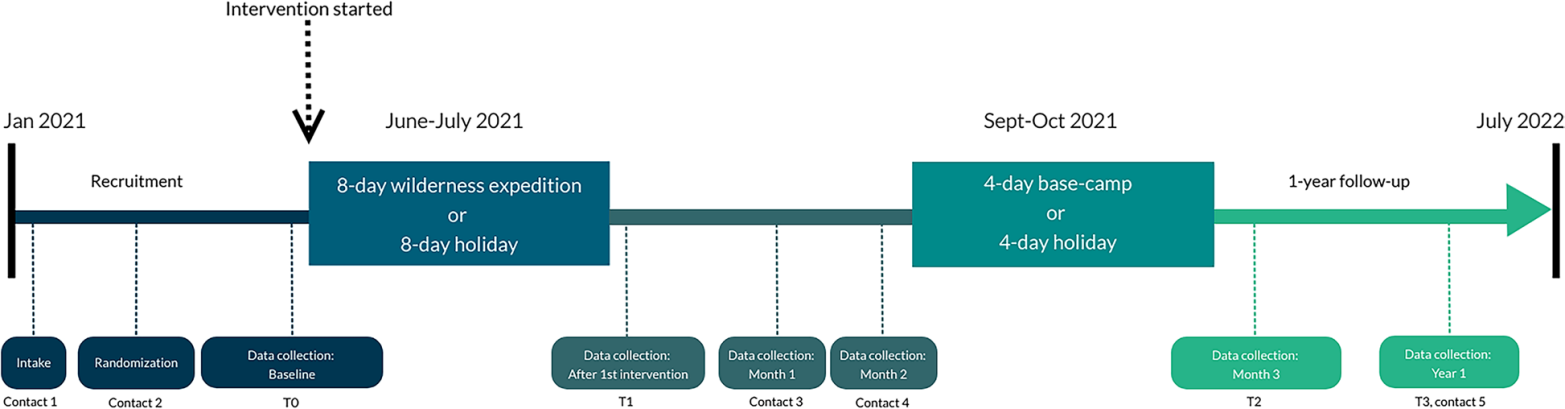
Schematic overview of the study

This study was a pilot RCT with a two-armed parallel-design using individual randomization. The protocol for this study was registered in a public trial registry (ClinicalTrials.gov: NCT04761042) and previously published [43]. The study was conducted according to the guidelines of the Declaration of Helsinki, and results are reported according to the Consolidated Standards of Reporting Trials (CONSORT), 25-item checklist and flow diagram [44]. Mid Sweden University (Sundsvall, Sweden) was the responsible body for the study, which took place in the county Västernorrland, Sweden from January 2021 to July 2022 (see Fig. 1).

### Participants and recruitment

Individuals eligible to participate were AYA’s aged 16–39 years who had received a cancer diagnosis during childhood, adolescence, or young adulthood. No eligibility restrictions were placed on the type of the individuals’ cancer diagnosis or time after cancer treatment. Participants had to be capable of walking at least 2 km without a pause, for which walking aids were allowed. Exclusion criteria was an active cancer treatment or medical condition that prevented safe travel and participation in the study, as evaluated by their treating physician/oncologist. Participants were recruited among a total of about 1983 childhood, AYA cancer survivors who were members of the Swedish cancer organization Ung Cancer (*n* = 1683) or the Swedish Childhood Cancer Fund (*n* = 300). A short announcement of the planned study was posted in e-newsletters and social media (Facebook) of these cancer organizations. The announcement was an open invitation for childhood, AYA cancer survivors aged 16–39 years to participate in a pilot study that aimed to compare participation in an outdoor program with participation in a holiday program regarding possible benefits for their health and well-being. Those interested were referred to the WAYA study website of Mid Sweden University [45] for more detailed information about the study and were asked to contact the last author (MJ). The purpose of the study was made clear to those interested in participating, as well as the voluntary nature of the study. Participation in the study could be stopped at any time without giving a reason, and individual data would be confidential. Written informed consent was obtained from all participants prior to study inclusion. Participants did not receive incentives for study participation. The first participant was enrolled (randomized) in the study on 26 January 2021. The last participant completed the study on 28 July 2022.

### Intervention arm: Content and structure of the WAYA program

The WAYA wilderness program was rooted in three fundamental components: 1) Theoretical foundation in the ecosophy of the Norwegian philosopher, mountaineer and environmental activist Arne Næss [46]; 2) Concept building using the Positive Health Model [47]; 3) Content building by means of a scoping review on other wilderness programs [41], and through patient and public involvement. The WAYA program aims were formulated around the six domains of Positive Health [47], and set to increase physical activity, self-confidence, self-efficacy and self-care, to support personal growth and building meaningful relationships, and to provide an enjoyable and safe stay in nature.

Nature in the WAYA program had both a contextual and active role in support of the health and well-being of childhood and AYA cancer survivors, wherein their health was regarded to be intrinsically interwoven in a bi-directional fashion with the health and well-being of nature [46]. Participants were given the opportunity to experience a diversity of natural landscapes, and specific nature characteristics were actively incorporated in program activities. Activities in the program included, among others, hiking, backpacking, sea-kayaking, rock climbing, camping, mapping/compass/orienting, trail cooking, safety skills training, equipment planning, foraging, fishing, bush-craft skills and leave no trace. The program also included (nature) reflective practices such as mindfulness, meditation, journaling, and forest bathing. All necessary outdoor clothing and equipment were made available for study participation, without any costs for participants. Upon participant’s request, the provided outdoor clothing and equipment could be borrowed after the conclusion of the program without any costs.

The structure of the wilderness program is graphically depicted in Fig. 1 and consisted of three intervention parts: 1) An eight-day wilderness expedition; 2) A three-month in-between period where participants were contacted twice to coach them to engage in their own outdoor activities; 3) and ended with a four-day base camp program. Both the expedition and base-camp program took place in nature settings around the High Coast of Sweden. The maximum group size was 10 participants, and the group structure was closed [28]. Facilitators in the WAYA program had outdoor, medical, counseling, supervision, and research competence. Three facilitators of the WAYA program were licensed health professionals, two nurses and a psychologist. The facilitator to participant ratio was 1:1. A more detailed description of the development and content of the WAYA program and the competences of the facilitators in this wilderness program has been published elsewhere [28].

### Comparison arm: Content and structure of a holiday program

Extensive discussions with researchers, patients and public representatives led to the design of a holiday program as an appropriate comparison arm. A holiday program was aimed to control for attention, group support and separation from home and job life, all factors that may positively influence the health and well-being of childhood and AYA cancer survivors [43]. Activities in the comparison arm allow the study to examine the impact of immersion in nature, challenging physical activities, meditation/mindfulness, and the group sense that grows from meeting challenges together.

Activities in the holiday program included, amongst others: Spa, fine dining, watching movies, reading, games, and visiting museums. Guided group gatherings took place twice a day (morning: check-in, evening: check-out) involving talking with and listening to how everyone was doing. The holiday program did not include guided nature, outdoor or structured physical activities.

Just like the WAYA program, the holiday program consisted of three parts: 1) An eight-day holiday; 2) A three-month period in between where participants were contacted twice to discuss how they were doing; and 3) A four-day holiday (Fig. 1). Participants stayed both times at the same Spa Hotel in the county of Västernorrland. The group size goal was ten participants, the group structure was closed, and the facilitator to participant ratio was 1:5. Facilitators had competence in guidance and supervision of groups, first aid, and research methodology.

### Study safety planning

A risk and safety plan was developed to assesses the potential risks and to describe the necessary precautions and preparations to perform the wilderness program with acceptable risks [28]. Since the study was performed between the 3rd and 4th wave of the COVID-19 pandemic in Sweden, participants and facilitators in the wilderness and holiday programs were tested upon arrival and at the first intervention day for COVID-19 by means of a Rapid Antigen Rapid Test Cassette (BioServ UK). Further COVID-19 testing was performed in case a participant or facilitator exhibited COVID-19 related symptoms. All participants and facilitators were covered by a study insurance under the intervention period. Two facilitators in the program were licensed nurses. Two medical doctors acted as remote advisors and could be contacted during program interventions if needed.

### Primary study outcomes

Primary study outcomes were study feasibility and safety. The following feasibility parameters were selected for this RCT: 1) *Time needed to recruit the participants*, calculated as the time (months) needed to recruit at least 40 eligible participants that were willing to be randomized. To obtain further insight regarding recruitment and inclusion of participants in the study, the following parameters were calculated: *Recruitment rate*: Calculated as the number of individuals who contacted Mid Sweden University expressing interest in the program compared with the estimated maximum number of individuals who could have read information on the study via Facebook and newsletters from the national cancer organizations; *Eligibility rate:* The number of individuals meeting the eligibility criteria divided by the total number of individuals who contacted Mid Sweden University; *Enrollment rate:* The number of individuals randomized into the study divided by the number of eligible individuals; 2) *Program preference and expectations.* Program preference (strong, light or no preference) and expectations (open question) were directly asked to eligible participants during the first contact; 3) *Willingness to be randomized*. This was asked to eligible participants at the first contact (yes/no) and calculated based on the number of participants that dropped out after randomization because they had hoped to be allocated to the other program or those that admitted after randomization that they would have dropped out if they had been allocated to the other program; 4) *Attrition rate before, and after start of the intervention:* Calculated as the number of individuals who dropped out of the study before and after study start, divided by the total number of enrolled participants; 5) *Adherence to the study program*, calculated as the number of intervention days on which participants attended the program-offered activities, divided by the total number of intervention days when program activities were offered; 6) *Data collection completeness*, calculated as the % of completed questionnaires divided by the total number (no.) of questionnaires to be completed as part of the study (including all time points).

Regarding safety, the occurrence of adverse events was closely monitored during the study interventions. An adverse event was defined as any incident, accident or another unfavorable or harmful outcome that occurred during or after the intervention but was not necessarily caused by the intervention [48]. In case of a certain, probable/likely, or possible causal relation between the adverse event and the program intervention, it was considered an *Adverse Effect (AE)* [48]. In both arms, adverse events were either reported by participants themselves during daily check-in and check-outs and/or reported by facilitators in their study field diary [28]. Verbatim descriptions (investigator terms from the field diaries and case report forms) of adverse events were translated to standardized medical terminology using the Medical Dictionary for Regulatory Activities (MedDRA) version 25.1 [49]. Adverse events were coded to the MedDRA preferred term (PT) and primary System Organ Class (SOC). Safety end points included the number (no.) and causality of adverse events, seriousness, outcome, severity, and type (PT and SOC) of AEs. Severity of AEs were graded according to the Common Terminology Criteria for Adverse Events (CTCAE) of the National Cancer Institute, version 4.03. Assessment of causality, outcome, and seriousness were performed by the study investigators and followed the guidelines and classifications of the WHO Uppsala Monitoring Centre [50]. Exposure to intervention was calculated for each treatment arm similarly to study program adherence as described above.

### Secondary study outcomes

Secondary study outcomes in this RCT were the following self-reported questionnaires:

*Nature Relatedness Scale (NRS):* A 21-item scale measuring ones ‘affective, cognitive, and physical relationship’ with nature [51]. Since the NRS was not available in the Swedish language, adequate translation, re-translation, and validation procedures were performed and results of that will be published elsewhere.

*Minneapolis Manchester Quality of Life instrument (MMQL)*, a seven quality-of-life domain questionnaire for AYA cancer survivors [52], validated in the Swedish language [53].

*Rosenberg Self-Esteem Scale (RSES),* a 10-item scale measuring global self-worth [54], and validated in the Swedish language [55].

*Generalized Self-Efficacy scale (GSE):* A 10-item scale measuring general belief in oneself to solve problems and reach goals [56], also translated and validated in the Swedish language [57].

Changes in secondary outcome measures were analyzed according to a pre-defined data analysis plan. Across all scales used to assess health-related outcomes, higher scores reflect more positive outcomes.

### Randomization and concealment

Participants were individually randomized to either the wilderness or holiday program by means of a Random Allocation Software Program using a random block size of two. Three age groups (16–19, 20–30, 31–39 years) and gender (male/female) were used as stratification factors to achieve an equal distribution between the two arms. Separate randomization lists were generated per strata. Allocation concealment was guaranteed since the randomization and assignment of participants to the two arms were performed independently by the first author (MCJ), who was not involved in participant recruitment. Although it was not possible to blind for the intervention, participants were informed that the study aimed to investigate two possible effective interventions, not revealing which intervention was hypothesized to be the most effective one. Furthermore, the statistical outcome analyses were performed by the second author (HD) who was blind to program allocation.

### Data collection and study procedures

Figure 1. gives an overview of the different timepoints at which data were collected. At contact moment 1, data were collected regarding obtained informed consent, participants willingness to be randomized, whether participants met the inclusion criteria, and preference and expectations of participants towards the two interventions. The following data were collected at contact moment 2: Demographic data and self-reported cancer characteristics, medical history, medication use, other treatments/therapies, and lifestyle measures (dietary, sleep, physical activity, stress, smoking, alcohol, time in nature). Self-reported health issues were coded to MedDRA PT. Possible changes in medication, treatment/therapies and lifestyle measures of participants were collected at contact moment 3–5, as well as whether any adverse events or other unexpected major event had occurred. To avoid either positive or negative effects related to pre-trip expectations or anxiety and post-trip enthusiasm, questionnaires data were collected 2–3 weeks before the start (T0) or 2–3 weeks after the 8-day intervention (T1) or 4-day intervention (T2). Data on possible changes in lifestyle and the occurrence of adverse events were collected at contact moment 3 (one month after the 8-day intervention), contact moment 4 (two months after the 8-day intervention), and contact moment 5 (one-year follow-up, T3). Questionnaire data were also collected at one-year follow-up. During the study, participants were allowed to continue or start their medications, therapies, diets, and lifestyle measures. Changes in any of these were documented in the case report form of each participant at the different contact moments. Detailed information on data management and confidentiality in the study has previously been published [43].

### Sample size and statistical analysis

We aimed to include a total of 40 participants, *n* = 20 in the wilderness program and *n* = 20 in the holiday program. This sample size was chosen following the recommendations of Whitehead et al. (37), to detect a small to medium effect size for changes in quality of life.

Safety analyses were performed on the safety analysis set and included all individuals that had participated in one of the program interventions for at least one day. A safety code book and a safety analysis plan assisted in the analysis. Crude (unadjusted) Relative Risk (RR) with 95% Confidence Intervals (CI) were calculated to indicate the risk of having an AE adjusted to days of exposure in program interventions. Pearson's chi-squared test was used to calculate differences in AE severity between the two study arms. Chi-squared statistics and independent samples t-tests were used to calculate group differences in socio-demographic variables and cancer characteristics. The Alpha level was set to 0.05.

To explore possible change over time in nature-relatedness, quality of life, self-esteem, and self-efficacy scores within study arms, linear mixed regression models with fixed effects were used. The covariance matrix was set to A1 Heterogenous which assumes that the covariance between time points gets weaker as measurement times grow further apart. To explore possible changes between study arms, a mixed-effects regression models with random intercepts were conducted. Missing data were imputed by means of multiple imputation [58]. Scores are depicted as mean ± SE with Akaike Information Criterion (AIC) for model estimations, and *p*-values < 0.05 to indicate significant differences. Statistical analyses were performed using SPSS Statistics version 28. Cohen’s d effect sizes were manually calculated using the mean difference (the beta) divided by the standard deviation, obtained from the standard deviation (SD) of the residuals.

## Results

### Recruitment and characteristics of participants

**Fig. 2:**
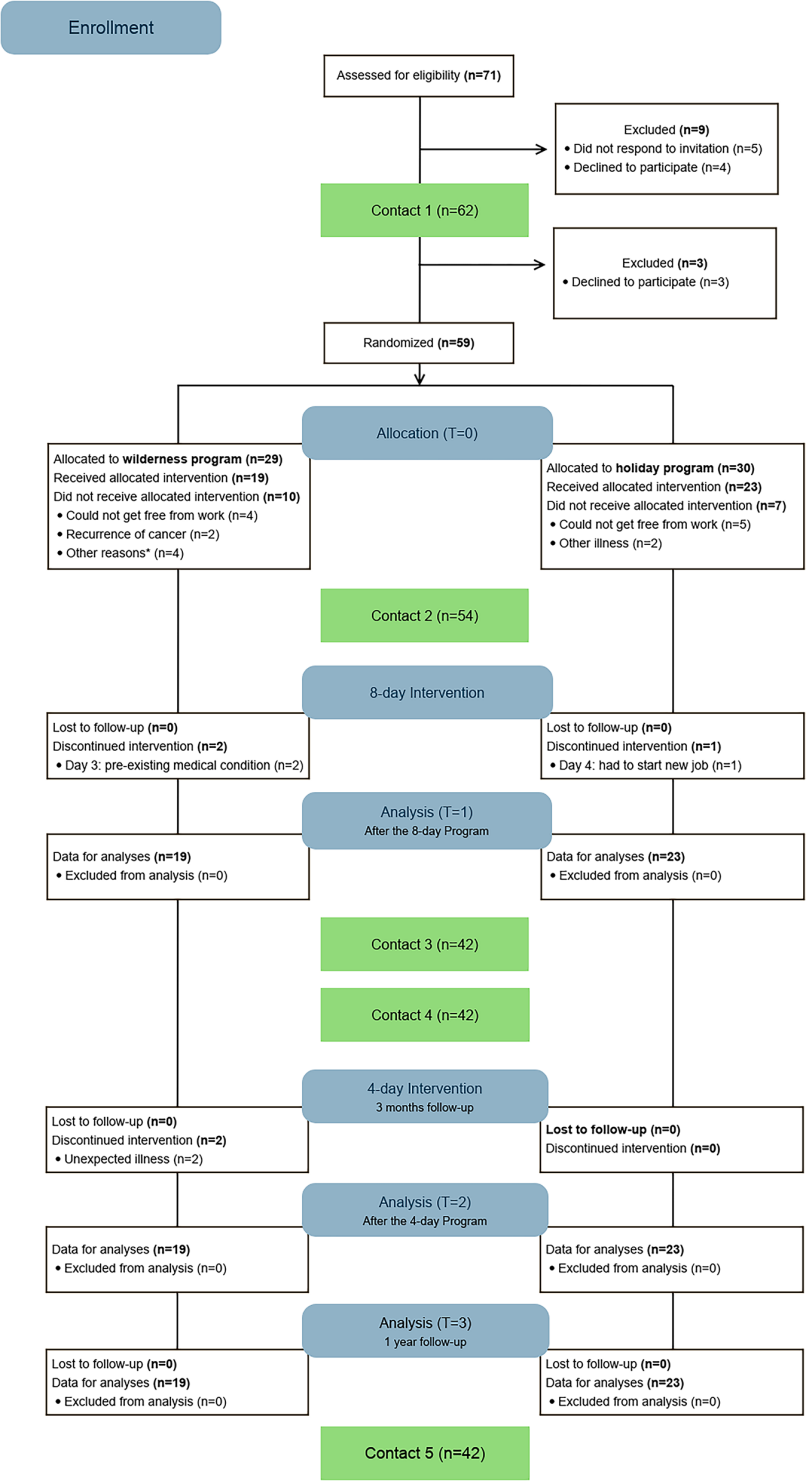
Consort flow diagram of participants in the study

The CONSORT flow diagram is depicted in Fig. 2. A total of 71 individuals contacted Mid Sweden University to receive more information about the WAYA study. Of those 71 that had showed initial interest, five individuals did not respond to an invitation for a first contact interview. Another four participants declined participation because they had either no time to engage in the study (*n* = 3), or because of health problems (*n* = 1). Therefore, a total of 62 individuals were screened at the first contact interview and met the eligibility criteria.

Out of these 62, three additional individuals declined participation due to time constraints (*n* = 2) or not wanting to be the youngest participant (16 years) in the study (*n* = 1). Ultimately, a total of 59 eligible individuals were randomized to either the wilderness program (*n* = 29) or holiday program (*n* = 30) (Fig. 2). Five out of 59 randomized individuals declined participation before they could be interviewed at contact moment two, mostly because they could not get free from work. A total of 54 individuals were therefore met at a second contact interview for collection of demographic data (baseline). Ten out of 29 individuals that were enrolled in the wilderness program did not receive the allocated intervention. Main reason was that they were not able to get free time from work. The same reason was also mentioned most often among the seven individuals that did not receive the holiday intervention. Therefore, a total of 19 individuals participated in the wilderness program, and a total of 23 individuals in the holiday program. The demographic and cancer characteristics of those that participated in the respective programs are presented in Table 1.

**Table 1:** Demographic and cancer characteristics of program participants at baseline

**Characteristics**	**Wilderness** *n* = *19*	**Holiday** *n* = *23*	*P*-value***
Age, mean ± SD	29.4 ± 3.8	29.1 ± 5.8	0.281
Gender, n (%)			
Male	7 (36.8)	5 (21.7)	
Female	12 (63.2)	18 (78.3)	0.830
Ethnicity, n (%)			
One or both parents born Sweden	18 (94.7)	23 (100)	
Both parents born outside Sweden	1 (5.3)	-	0.265
Marital status, n (%)			
Married-Partner	9 (47.4)	7 (30.4)	
Single	10 (52.6)	16 (69.6)	0.261
Family situation, n (%)			
No children	18 (94.7)	21 (91.3)	
Children	1 (5.3)	2 (8.6)	0.651
Education, n (%)			
Comprehensive	1 (5.3)	1 (4.3)	
High School	6 (31.6)	5 (21.7)	
College/university	10 (52.6)	15 (65.2)	
Other	2 (10.5)	2 (8.7)	0.869
Employment status, n (%)			
Full time work	9 (47.4)	4 (17.4)	
Part time work	4 (21.1)	10 (43.5)	
Unemployed	-	1 (4.3)	
Full time education	2 (10.5)	4 (17.4)	
Unable to work	4 (21.1)	4 (17.4)	0.212
Relative economic situation^a^, n (%)			
Always	9 (47.4)	9 (39.1)	
Often	8 (42.1)	10 (43.5)	
Sometimes	1 (5.3)	1 (4.3)	
Seldom	1 (5.3)	3 (13.0)	0.838
Primary cancer type, n (%)			
Leukemia	5 (26.3)	7 (30.4)	
Brain	5 (26.3)	6 (26.1)	
Lymphoma	1 (5.3)	4 (17.4)	
Neuroblastoma	-	1 (4.3)	
Soft tissue sarcoma	-	1 (4.3)	
Bone	1 (5.3)	-	
Breast	2 (10.5)	-	
Testicular	2 (10.5)	1 (4.3)	
Endocrine	-	1 (4.3)	
Thyroid	2 (10.5)	2 (8.7)	0.230
Epipharynx	1 (5.3)	-	
Age at cancer diagnosis, mean ± SD (min–max)	19.1 ± 8.4 (1.5–29)	12.5 ± 9.4 (0–29)	**0.024**
Survivorship, n (%)			
Childhood cancer	5 (26.3)	12 (52.2)	
AYA cancer	14 (73.7)	11 (47.8)	0.089
Cancer treatment^b^, n (%)			
Surgery	12 (37.5)	14 (36.8)	
Chemotherapy	11 (34.4)	15 (39.5)	
Radiotherapy	8 (25.0)	8 (21.1)	
Hormone therapy	1 (3.1)	1 (2.6)	
Time since last treatment, n (%)			
< 3 months	4 (21.1)	-	
4–11 months	1 (5.3)	3 (13.0)	
1 year—5 years	5 (26.3)	5 (21.7)	
> 5 years	8 (42.1)	14 (60.9)	
On active treatment/surveillance	1 (5.3)	1 (4.3)	0.177
Medication use, n (%)			
Any medication	16 (84.2)	17 (74.0)	
Number of types of medications	43	29	
Mean number of medications ± SD	2.6 ± 2.0	1.8 ± 1.6	0.692
Most frequent type of medication:			
1. Hormone, no. (%)	16 (37.2)	18 (62.1)	
2. Pain, no. (%)	6 (14.0)	-	
3. Anti-depressants, no. (%)	5 (11.6)	6 (20.7)	

^a^Relative economic situation was formulated as: How often do you have good finances to be able to do the same things as your friends

^b^Cancer treatment, more than one treatment possible

^***^ Chi-2 statistics for nominal and ordinal data and independent samples t-tests for interval/ratio data

Participants’ mean age was 29 years and the majority in both programs was female with both parents born in Sweden. Participants varied with respect to primary cancer type, but most common cancer diagnoses in both study arms were leukemia and brain tumor (Table 1). The wilderness and holiday program participants were quite similar in demographic characteristics, with the only exception of age at cancer diagnosis. Participants in the wilderness program were significantly older at cancer diagnoses than those in the holiday program (19.1 ± 8.4 versus 12.5 ± 9.4, *p* < 0.024). This was reflected by a higher percentage of AYA cancer survivors in the wilderness compared to the holiday program (73.3 versus 47.8%, respectively) (Table 1). At baseline, the majority of participants in both programs was using one or more types of medication, with an average number of 2.6 in the wilderness and 1.8 in the holiday arm (Table 1). Most frequent type of medications used by participants in the wilderness program were hormone related (37.2%) such as thyroid, sex or pituitary hormones (no. = 9), anti-conception (no. = 4) or corticosteroids (no. = 3). Other frequently used medications were pain medication (14.0%), such as NSAIDs (no. = 3) and other analgesics (no. = 3), and anti-depressants (11.6%) (Table 1). Hormone-related medications were also most frequently used by participants in the holiday program (62.1%), including sex hormones (no. = 8), thyroid hormones (no. = 6) or anti-conception (no. = 4). Anti-depressant medication added up to 26% of all medications used in the holiday program. In contrast to participants in the wilderness program, the participants in the holiday program did not use any standard pain medication (Table 1).

The demographic characteristics of the 12 randomized participants that were met at contact moment two but did not receive the intervention (*n* = 7 in the wilderness arm and *n* = 5 in the holiday arm), did not significantly differ from those that received the allocated interventions (*p*-values from 0.069–0.977).

All 19 participants in the wilderness program reported suffering from long-term health issues, with an average of 3.3 health issues per participant. As shown in Table 2, the most frequently self-reported health issues were joint pain, allergies, mild neurocognitive disorders, depression, and other mental disorders. In the holiday program, 22 out of 23 participants (95.7%) reported an average of 3.8 health issues. The most frequently self-reported long-term health issues among participants in the holiday program were mild neurocognitive disorders, hearing or visual impairment, allergies, pain, and anxiety (Table 2).

**Table 2:** Self-reported long-term health issues of program participants at baseline by study arm

**Health issue**^a^	**Wilderness** *n (%)*	**Holiday** *n (%)*
Mild neurocognitive disorder	5 (26.3)	12 (52.2)
Hearing impaired	3 (15.8)	9 (39.1)
Joint pain	11 (57.9)	1 (4.3)
Allergy	6 (31.6)	6 (26.1)
Pain	2 (10.5)	6 (26.1)
Depression	5 (26.3)	4 (17.4)
Anxiety	2 (10.5)	5 (21.7)
Memory impaired	1 (5.3)	4 (17.4)
Fertility decreased	-	3 (13.0)
Mental disorders	5 (26.3)	1 (4.3)
Visual impairment	2 (10.5)	7 (30.4)
Social phobia	-	2 (8.7)
Numbness	-	2 (8.7)
Tiredness	2 (10.5)	1 (4.3)
Balance difficulty	2 (10.5)	2 (8.7)
Sleep disorders	2 (10.5)	-
Gastrointestinal motility disorder	2 (10.5)	2 (8.7)
Eating disorder	-	2 (8.7)
Migraine	-	2 (8.7)
Concentration impairment	1 (5.3)	1 (4.3)
Joint range of motion decreased	1 (5.3)	-
Paresis	1 (5.3)	-
Gout	1 (5.3)	-
Gluten intolerance	1 (5.3)	-
Osteoporosis	1 (5.3)	-
Increased skin sensitivity	1 (5.3)	-
Other disorders of neurohypophysis	1 (5.3)	1 (4.3)
Asthma	1 (5.3)	-
Lactose intolerance	1 (5.3)	1 (4.3)
Anemia	1 (5.3)	-
Hormonal imbalance	1 (5.3)	1 (4.3)
Congenital heart disease NOS	-	2 (8.7)
Stress symptoms	-	1 (4.3)
Asperger's syndrome	-	1 (4.3)
Vitamin D deficiency	-	1 (4.3)
Mood swings	-	1 (4.3)
Sinusitis	-	1 (4.3)
Kidney dysfunction	-	1 (4.3)
Drug use issue	-	1 (4.3)

^a^Standardized to medical terminology of MedDRA®

### Primary outcomes

Primary outcomes of this study were a set of feasibility parameters related to performing a RCT, as well as the safety of the wilderness and holiday program. Feasibility outcomes are listed in Table 3, and safety end points in Tables 4 and 5.

**Table 3:** Study feasibility parameters

Feasibility parameter	Total	Wilderness	Holiday
Time needed to recruit, months	6	-	-
Recruitment rate, %	3.6	-	-
Eligibility rate, n (%)	62 (87.3)	-	-
Total	71 (100)	-	-
Enrollment rate, n (%)	59 (95.2)	-	-
Total	62 (100)	-	-
Program preference, n (%)			
Wilderness program	36 (58.1)	18 (62.1)	18 (60.0)
Holiday program	11 (17.7)	4 (13.8)	6 (20.0)
No preference	15 (24.2)	7 (24.1)	6 (20.0)
Total	62 (100)	29 (100)	30 (100)
Willingness to be randomized, n (%)			
Yes	60 (96.8)	28 (96.6)	29 (96.7)
No	2 (3.2)	1 (3.4)	1 (3.3)
Total	62 (100)	30 (100)	30 (100)
Attrition rate before study start, n (%)	17 (28.8)	10 (34.5)	7 (23.3)
Total	59 (100)	29 (100)	30 (100)
Attrition rate after study start, n (%)	0 (0)	0 (0)	0 (0)
Total	42 (100)	19 (100)	23 (100)
Adherence to study program, days (%)	479 (95.0)	209 (91.7)	270 (97.8)
Total	504 (100)	228 (100)	276 (100)
Data collection completeness, no. (%)	660 (98.2)	294 (94.7)	366 (99.5)
Total	672 (100)	304 (100)	368 (100)

**Table 4:** Characteristics of adverse effects (AEs)

**Characteristics**	**Total** n / no. (%)	**Wilderness** n / no. (%)	**Holiday** n / no. (%)	**RR** (CI)
Adverse events	42 / 294 (100)	19 / 118 (40.1)	23 / 176 (59.9)	1.2 (0.9–1.5)
Causality, no. (%)				
Certain	41 (13.9)	34 (28.8)	7 (4.0)	6.2 (4.1–9.3)
Probable/likely	40 (13.6)	28 (23.7)	12 (6.8)	3.0 (2.2–4.2)
Possible	173 (58.8)	51 (43.2)	122 (69.3)	1.9 (1.7–2.2)
Unlikely/remote	40 (13.6)	5 (4.2)	35 (19.9)	5.4 (3.5–8.3)
Conditional/unclassified	0	0	0	-
Unassessable	0	0	0	-
AEs	39 / 254 (100)	17 / 113 (44.5)	23 / 141 (55.5)	1.0 (0.8–1.3)
Seriousness AEs				
Non-serious	39 / 254 (100)	17 / 113 (44.5)	23 / 141 (55.5)	1.0 (0.8–1.3)
Serious	0	0	0	-
Outcome AEs				
Fatal	0	0	0	-
Not recovered/resolved	0	0	0	-
Recovered/resolved	39 / 254 (100)	17 / 113 (44.5)	23 / 141 (55.5)	1.0 (0.8–1.3)
Recovered with sequelae	0	0	0	-
Sequelae	0	0	0	-
Worsening	0	0	0	-
Unknown	0	0	0	-
Severity AEs				
Grade 1	36 / 104 (40.9)	14 / 43 (38.1)	22 / 61 (43.3)	1.1 (0.9–1.3)
Grade 2	36 / 150 (59.1)	15 / 70 (61.9)	20 / 80 (56.7)	1.1 (1.0–1.3)
Grade 3–5	0	0	0	-
Program part AEs				
Expedition / Holiday	39/ 194 (6.3)	17 / 92 (81.4)	22 / 102 (72.3)	1.2 (1.1–1.2)
Base-camp / Holiday	29 / 60 (23.6)	10 / 21 (18.6)	19 / 39 (27.7)	1.4 (1.2–1.5)
Participant gender				
Female	30 / 191 (75.2)	12 / 73 (64.5)	18 / 118 (83.7)	1.0 (0.9–1.1)
Male	10 / 63 (24.8)	5 / 40 (35.4)	5 / 23 (16.3)	1.2 (1.1–1.4)
Participant age				
20–30 years	26 / 168 (66.1)	12 / 83 (73.5)	14 / 85 (60.3)	1.1 (1.0–1.2)
31–39 years	14 / 86 (33.9)	5 / 30 (26.5)	9 / 56 (39.7)	1.0 (1.0–1.2)
Survivorship				
Childhood cancer	17 / 151 (51.4)	5 / 32 (27.1)	12 / 119 (67.6)	1.5 (1.4–1.6)
AYA cancer	23 / 143 (48.6)	12 / 86 (72.9)	11 / 57 (32.4)	1.1 (1.1–1-2)

**Table 5:** Most frequently reported adverse effects (AEs) by Preferred Term (PT) and System Organ Class (SOC)

Wilderness		Holiday	
**PT**	**n / no. (%)**	**PT**	**n / no. (%)**
1.Tiredness	11 / 22 (19.5)	1.Tiredness	17 / 56 (39.7)
2.Insect bite	5 / 9 (8.0)	2.Headache	9 / 19 (13.5)
3.Situational anxiety	6 / 9 (8.0)	3.Sleeplessness	9 / 9 (6.4)
4.Constipation	3 / 6 (5.3)	4.Insomnia	7 / 8 (5.7)
5.Tension headache	1 / 6 (5.3)	5.Mild neurocognitive disorder	6 / 8 (5.7)
**SOC**	***n / no. (%)***	**SOC**	***n / no. (%)***
1.General disorders and administration side conditions	12 / 25 (22.1)	1.General disorders and administration side conditions	17 / 59 (41.8)
2.Psychiatric disorders	12 / 25 (22.1)	2.Psychiatric disorders	15 / 33 (23.4)
3.Injury, poisoning and procedural complications	10 / 24 (21.2)	3.Nervous system disorders	12 / 29 (20.6)
4.Musculoskeletal and connective tissue disorders	9 / 16 (14.2)	4.Musculoskeletal and connective tissue disorders	8 / 9 (6.4)
5.Gastrointestinal disorders	7 / 11 / (9.7)	5.Ear and labyrinth disorders	3 / 3 (2.1)

### Feasibility

Time needed to recruit the participants: The time needed to recruit at least 40 eligible participants in the study was six months (January-June 2021) (Table 3). The number of potentially eligible cancer survivors that were exposed to study recruitment efforts of the two Swedish cancer organizations through their membership was estimated to be 1983. A total of 71 individuals expressed interest in the study at Mid Sweden University, resulting in a recruitment rate of 3.6% (Table 3). All 62 individuals that were screened at contact 1 met the eligibility criteria. Therefore, the eligibility rate was 87,3%. Since 59 out of 62 eligible participants were randomized to either the wilderness or holiday program, the enrollment rate was 95.2% (see Table 3).

Program preference and expectations: Out of 62 individuals that were screened for eligibility during the first contact, most (58.1%) preferred to participate in the wilderness program (Table 3). In 17.7% of the cases the holiday program was preferred, and 24.2% (*n* = 15) of individuals had no preference for either one. Preference of participants for either the wilderness or holiday program did not significantly differ between the two arms in the study (*p* = 0.426). Before study start, individuals expected the wilderness program to be challenging (*n* = 24), a place to meet and talk with others that have gone through the same (*n* = 19), to experience something new (*n* = 17), and to get energy from being (physically active) out in nature (*n* = 17). Expectations about the holiday program were mostly time to relax (*n* = 47), to meet and talk with others (*n* = 26), nice to be away (*n* = 10), risk to get bored (*n* = 9), and time for reflection (*n* = 5).

Willingness to be randomized: Although all 62 individuals agreed to be randomized at the first contact, it turned out that one individual who had a strong preference for the holiday program dropped out of the study because she had been allocated to the wilderness program. Another participant who ended up in the holiday program confessed that if she had been randomized to the wilderness program, she would have dropped out. Willingness to be randomized was thus 60 out of 62 individuals (96.8%) (Table 3).

Attrition rate: Out of the 59 individuals initially enrolled, a total of 17 dropped out after randomization but before start of the intervention. The attrition rate before the start of the intervention was thus 28.8% in total, 34.5% (10/29) in the wilderness and 23.3% (7/30) in the holiday program. Since no participant dropped out of the study after entering the intervention, neither in the wilderness program nor in the holiday program, the attrition rate after start of the intervention was 0% overall and, in both programs (Table 3).

Adherence to the study program: The adherence to program-related activities was 91.7% in the wilderness program, and 97.8% in the holiday program (Table 3). During the wilderness expedition with the first group, two out of ten participants discontinued the program after three days due to a pre-existing medical condition [28]. Furthermore, two out of ten participants in the first wilderness group did not participate in the base-camp intervention, due to a suspected COVID-19 related illness (*n* = 1) and an unexpected illness in the family (*n* = 1). In the holiday program, one participant in the first group discontinued intervention after four days because she suddenly had to start a new summer job (Fig. 2).

Data collection completeness: There were few missing data in both arms. Overall, 96.7% of all questionnaires were completed by participants in the wilderness program and 99.5% of all questionnaires by participants in the holiday program (Table 3). Data collection completeness at one year follow up was also high, 94.7% (72/76 completed questionnaires) in the wilderness and 98.9% (92/93 completed questionnaires) in the holiday arm.

### Safety end points

During the whole study, a total no. of 294 adverse events were reported (see Table 4). Most of them (*n* = 254) were labeled as AEs since they were certain, probable/likely, or possibly related to one of the program interventions. In the wilderness program, 17 out of 19 participants reported a total of 113 AEs. In the holiday program all participants (*n* = 23) reported at least one AE, with a total of 141 AEs during the whole study period. The RR for the occurrence of AEs in the wilderness program was not significantly higher than in the holiday program (RR: 1.0 (CI 0.8–1.3)) (Table 4).

AEs were of mild or moderate severity (grade 1–2), all AEs were resolved during the study period (Table 4) and no severe AEs were reported during either of the programs. The RR for reporting moderate AEs was slightly but significantly higher in the wilderness versus the holiday program (1.1, CI: 1.0–1.3). As shown in Table 4, the majority of AEs was reported by female participants (*p* < 0.001) in the younger age group (20–30 years, *p* < 0.03). Slightly higher RR with significant differences were observed between the two study arms for the variables program part, gender, age, and survivorship (Table 4). However, no clear pattern was identified either in favor or against the wilderness or holiday program.

As shown in Table 5, tiredness was the most frequently reported AE by participants during both programs. Other frequently AEs reported during the wilderness program were mosquito (insect) bites, situational anxiety, constipation, and tension headache (*n* = 1). Frequently reported AEs by participants in the holiday program were problems to fall asleep (sleeplessness), to stay asleep (insomnia) and specific brain fatigue (mild neurocognitive disorder). The most frequent MedDRA SOC groups of AEs in both the wilderness and holiday program were general disorders and administration site conditions and psychiatric disorders (Table 5). The listing of all reported AEs can be found in Supplementary File 1 (S1: AE listing WAYA study).

### Secondary outcomes

This feasibility study was not powered to test any hypothesis regarding effectiveness of the wilderness program on the health and well-being of childhood and AYA cancer survivors. Statistical analysis of secondary outcome variables was therefore only exploratory as to indicate possible changes and trends. As shown in Table 6, the NRS increased over time upon participation in the wilderness program (AIC: 110.35, *p* < 0.001). No significant changes over time were observed among participants in the holiday program (AIC: 43.77, *p* = 0.560). *P*-values of within study arm differences are not shown in the Table 6. In-between arm differences were significantly different after one year (T3) compared to baseline (T0), meaning that participants in the wilderness program had a significantly higher average increase in NRS between the measurement points compared to the holiday program (AIC: 101.44, *p* < 0.001) with a medium Cohen's d effect size of 0.63 (Table 6).

**Table 6:** Nature-relatedness (NRS), quality of life (MMQL), self-esteem (RSES), and self-efficacy (GSE) outcomes

**Outcome**	**Wilderness** *n* = 19	**Holiday** *n* = 23	***P*-value**^a^	**Effect size**^**b**^
NRS, mean ± SE				
T0 = baseline	3.67 ± 0.14	3.76 ± 0.14		
T1 = after 8-day intervention	3.94 ± 0.15	3.80 ± 0.15		
T2 = 3 months	3.95 ± 0.15	3.78 ± 0.14		
T3 = 1 year	4.10 ± 0.13^c^	3.80 ± 0.14	** < 0.001**	0.63
MMQL, mean ± SE				
T0 = baseline	3.47 ± 0.11	3.23 ± 0.09		
T1 = after 8-day intervention	3.57 ± 0.13	3.24 ± 0.10		
T2 = 3 months	3.66 ± 0.13	3.26 ± 0.10		
T3 = 1 year	3.63 ± 0.13	3.30 ± 0.08	0.272	0.21
RSES, mean ± SE				
T0 = baseline	28.68 ± 1.36	26.35 ± 1.26		
T1 = after 8-day intervention	28.47 ± 1.07	26.26 ± 1.23		
T2 = 3 months	29.21 ± 1.31	26.00 ± 1.31		
T3 = 1 year	29.36 ± 1.63	27.51 ± 1.21^d^	0.900	0.02
GSE, mean ± SE				
T0 = baseline	2.99 ± 0.09	2.74 ± 0.09		
T1 = after 8-day intervention	2.95 ± 0.11	2.69 ± 0.09		
T2 = 3 months	2.96 ± 0.11	2.70 ± 0.09		
T3 = 1 year	3.10 ± 0.13	2.87 ± 0.09^e^	0.876	0.02

^a^Mixed-effects regression analysis with random slopes indicating differences between study arms

^b^Cohen's d effect size

^c^Indicating significant differences within study arm at T3 vs T0

^d^Indicating significant differences within study arm at T2 versus T3. ^e^Indicating significant differences within study arm at T1 and T2 vs T3

No significant differences between the two study arms were observed regarding health-related quality of life (MMQL), self-esteem (RSES) and self-efficacy (GSE) (Table 6). Further analysis of the seven sub-domains within MMQL showed that participants in the wilderness program had a significantly higher average increase in MMQL cognitive functioning scores over time than participants in the holiday arm (AIC 258.33, *p* = 0.031). No differences between study arms were observed for the other MMQL domains (results not shown). No significant differences in MMQL, RSES or GSE scores over time were found at any time-point within the wilderness program. There were significantly lower RSES scores at T2 compared to T3 within the holiday program (AIC 499.89, *p* = 0.030), and significantly lower GSE scores at T1 (AIC 45.81, *p* = 0.021) and T2 (AIC 45.81, *p* = 0.009) compared to T3 within the holiday program. MMQL scores over time within the holiday program were not significantly different (Table 6).

## Discussion

### Main findings

The present study shows that it is feasible to perform a randomized-controlled study design on a complex intervention such as a wilderness program and with a medically vulnerable study group such as childhood and AYA cancer survivors. Within half a year, the number of participants needed for this pilot study was recruited and almost all childhood and AYA cancer survivors were willing to be randomized. These results are thus in sharp contrast to the previous study by Gabrielsen et al. [42], where it appeared not feasible to perform such an RCT on wilderness therapy among adolescents with mental health problems [42]. Most likely the choice of the comparison arm in the present study explains why our RCT design was successful, i.e. the choice of the holiday program seems to increase willingness of survivors to approach the study group, to be randomized, and willingness to stay in the assigned arm. Gabrielsen et al. [42] intended to investigate a group undergoing wilderness therapy compared to a group receiving treatment as usual. Subsequently, adolescents in the control group started to drop-out because they were disappointed not to be randomized to the ‘active’ program, even if they could receive wilderness therapy after the study. In the present study, participants in the comparison arm were not doing ‘nothing’ (wait-list control) or ‘only’ received care as usual but were offered another and apparently for them, interesting program. Despite the fact that most participants originally preferred to participate in the wilderness program, the attrition rate after randomization was lower in the holiday arm compared to the wilderness arm (23.3% versus 34.5%, respectively). The choice for the holiday program as a comparison arm was inspired by a previously published study design aiming to investigate the effects of meditation versus vacation effects on aging-related biomarkers [59]. It is demonstrated that going on vacation may have positive effects by reducing fatigue and improving well-being both during the vacation and directly in the days thereafter [60, 61]. However, these positive effects rapidly decline upon returning home and are back within 1–3 weeks to the levels that they were before the vacation [60, 62]. The aims of the wilderness program intervention on the other hand, were to engage participants in nature activities and to increase their self-efficacy in support of their well-being in the longer term. It was therefore investigated whether it would be feasible to collect outcome data over a longer period of time, i.e. up till one year after the start of the intervention. The attrition rate after start of the first intervention was 0% in both programs, and data completions after one year were high in both programs (> 94%). The present study thus showed that it is feasible to collect good quality data over a one-year period among this cancer survivor group. It has previously been reported that it is difficult to recruit and/or retain young cancer survivors in clinical trials research [21]. Therefore, the positive feasibility outcomes as achieved in the present study suggest that the wilderness program intervention is developmentally appropriate and of interest for this population.

Another important finding of this study is that a supervised wilderness adventure is just as safe for childhood and AYA cancer survivors as enjoying a holiday program at a Spa hotel. The relative frequency of reported AEs was similar in both study arms, and all AEs were non-serious, mild, or moderate of severity, and resolved during the study period. Reports of tiredness, mosquito bites, situational anxiety, and constipation by participants in the wilderness program is to be expected when exposed to a wilderness expedition, challenging physical activities such as sea-kayaking and rock climbing, and outdoor food and no access to a bathroom. Despite the taxing wilderness conditions, it was interesting that tiredness was also frequently reported by participants in the holiday program. Whereas participants in the wilderness program had full days of physical activity in nature, participants in the holiday program were mostly relaxing, sleeping and enjoying good food. It may be that more vigorous outdoor physical activity resulted in higher quality sleep, increased group connectedness, and greater well-being, albeit, not statistically different between study programs. It is noted that sleeplessness and insomnia were more often reported by participants in the holiday program.

Although AEs were commonly reported by participants during both programs, it should be noted that several of these were to a greater or lesser extent related to their existing long-term health issues prior to study start (see Table 2). For example, pre-existing mild neurocognitive disorders, pain, tiredness, or anxiety were reported by participants to increase upon exposure to other hotel guests in the holiday program. Other every-day life events may trigger similar exacerbation of these long-term and late effects among childhood and AYA cancer survivors.

To the best of our knowledge, this is the first systematic and thorough analysis on the safety of wilderness programs for childhood and AYA cancer survivors. In a previous scoping review, we identified that only two out of the 15 included wilderness studies reported some program safety-related data [41]. A study by Dasson [63] reported on insect bites, headaches, stomach aches, and small injuries upon participation in a nature cancer camp, but did not further quantify or analyze these AEs. Similarly, minor injuries among childhood cancer survivors were observed upon participation in a ski-rehabilitation program, without further quantifying or elaborating their significance [64]. The lacuna in safety aspects of wilderness programs for young cancer survivors is surprisingly persistent. Recent systematic reviews and meta-analysis on the effectiveness of adventure and wilderness programs among AYA cancer survivors [29] or juvenile delinquents [65] do not mention nor discuss program-related safety issues. Safety reporting is also lacking in another recently published study on the effects of a wilderness program among AYA cancer survivors [66]. In clinical guideline development and other health-related decision making, possible benefits of wilderness programs should always be weighed against the risks of participation in such programs. Safety data also assist in the a-priori development of program safety planning necessary to perform the program with acceptable risks and to prevent AEs where possible. The safety analysis of the present study therefore provides valuable information for wilderness program optimization and future study planning.

Exploratory analyses of secondary outcomes pointed towards increased nature connectedness among participants in the wilderness program. The construct of nature connectedness measured in this study captured differences in the way individuals view their relationship with the natural world [51]. The wilderness program in the present study explicitly aimed to provide childhood and AYA cancer survivors with joy, balance, and safety within the grandness of nature [28] with the theory that nature provides peace, comfort, a feeling of freedom or an expression of spirituality [67, 68]. Since program activities such as mindfulness exercises were intended to support participants in building their relationship with nature, it was to be expected that we found significant between-group difference in nature relatedness in favor of the wilderness group. Previous studies have reported on positive associations between nature connectedness and mental well-being [69–71] and demonstrated that individuals with increased nature connectedness have greater sense of eudemonic well-being [72]. The exploratory statistical analyses in the present study did not point towards differences in quality of life and mental well-being of participants in the wilderness versus the holiday program. However, the present study was not designed nor powered to investigate possible significant differences in health outcomes between the two study arms.

Previous non-randomized controlled studies have reported that wilderness programs improve health and well-being of childhood and AYA cancer survivors [73–75]. A 10-day outdoor expedition program, Tip of the Toes, in Canada significantly improved self-esteem among 14–20 year cancer survivors directly after and up to one year after program participation [73]. Similarly, self-esteem of AYAs was significantly increased after participation in a six-day adventure program compared to a wait-list control group [74]. Another study demonstrated significant improvements in self-efficacy of AYA cancer survivors after participation in a one-week outdoor adventure program [75]. A direct comparison of those study outcomes with the finding in the present study is not possible due to differences in applied scales and constructs of self-esteem and self-efficacy, as well as a lack of detailed numerical outcome data in these previously published studies [73–75].

### Strengths and limitations

A strength of the present study is that it is the first attempted RCT on a wilderness program for cancer survivors. Previous studies have investigated health effects of such programs among young adult cancer survivors, but in non-randomized and controlled study designs [74–76]. Furthermore, several RCTs have been performed in Hong Kong on the effectiveness of adventure therapy among childhood and AYA cancer survivors, but these interventions did not involve immersion into nature and wilderness [77–79]. Other strengths of this study were that it included a long-term follow-up, that all participants in both arms after start of the actual intervention remained in the study, and the high percentage of data collection completeness through one year.

Since this was a feasibility study with low power due to a small sample size, it is obvious that no conclusions can be drawn about the effectiveness of a wilderness program on the health and well-being of childhood and AYA cancer survivors. A limitation of the present study design was that it did not investigate the feasibility of including a third arm in the study that received no intervention, for example a wait-list control. Based on the previous RCT experience of the Norwegian research group [42], we regarded it as unethical to make another attempt on the feasibility of including a wait-list control. Another limitation of the present study is that the two distinct cancer survivor age-cohorts, childhood and AYA cancer survivors, were grouped in the exploratory analysis of self-reported health outcomes. Since the age at first cancer diagnoses was significantly different at baseline between the wilderness and holiday arm, it is recommended to stratify by childhood or AYA cancer survivorship in future larger trials and possibly perform specific sub-group analysis regarding health-related outcome parameters. Furthermore, we are well aware of the fact that the study intervention took place during the COVID-19 pandemic and that this has impacted the outcome. Before study start, the majority of participants had not been socially active, joined group activities or travelled for vacation for greater than a year because of COVID. Therefore, participants in both groups reported positive experiences to meet and interact with others again after such a long time. However, at the same time it was reported, specifically among participants in the holiday program, that it was challenging to be around so many people with respect to increased exposure to noise and interactions than they had gotten used to.

### Considerations and recommendations for further research

The purpose of this study was to lay the foundation for a future larger RCT investigating the effectiveness and safety of a wilderness program on the health and well-being of childhood and AYA cancer survivors. Although the time to recruit the participants for this pilot study was relatively short (six months), it appeared that the overall recruitment rate was very low (3.6%). It is likely that the number of cancer survivors exposed to the study outreach was much lower than the total number of people who were signed up with the two cancer organizations. Furthermore, response may have been lower due to COVID-19 concerns. Other recruitment strategies to supplement members of national cancer organizations may be needed in order to reach out to this population. Health care, educational and cultural systems are quite similar in the Nordic countries. It is therefore recommended to perform a future larger multi-center RCT in close collaboration with centers in Denmark, Finland, and Norway. Another recommended strategy for recruitment of participants is to collaborate with hospitals in future trials.

Another important observation was that a substantial percentage of eligible individuals dropped out before study start (attrition rate 28.8%) because of lack of time or not being able to get free from work. Previous studies have published difficulties in recruiting AYA cancer survivors [21, 26]. This age group is at a stage of life with less job predictability and less security in the work world. In fact, AYA cancer survivors have less secure economic status than their age-group without cancer [80, 81]. Thus for example when offered a summer job or if participation increases risk losing a job, they clearly had to prioritize work.

It was intended to recruit both adolescent and young adults for participation in the present pilot study. To our surprise, it was only young adults from 20 years and older that participated. Only one eligible adolescent showed interest to participate but declined participation when she found out that she would be the youngest in the group. Since both national cancer organizations through which participants were recruited have adolescents among their members, it appears that such programs mainly appeal to young adults. The WAYA program was specifically developed for AYAs aged 16 to 39 years. This allowed for participation of individuals that were diagnosed with cancer throughout childhood, adolescents, and young adulthood. We previously reported that inclusion of a diverse group of young cancer survivors in a wilderness program seems to be of added value for group bonding and learning purposes [28]. The content of this wilderness program was found to be acceptable for both childhood and AYA cancer survivors [28]. Based on this previously published qualitative evaluation of the wilderness program we recommend: optimizing the program by decreasing the hiking pace at the expedition start; offering warmer sleeping bags; more fresh food: and at least two different options for daily expedition routes [28]. The observations that no participant dropped out after study start in the holiday program and that adherence to the holiday program was high (97.8%), demonstrates that the holiday program is also well accepted by childhood and AYA cancer survivors. The only recommendation for future studies related to improvement of the holiday program, is to omit bowling from the activity program due to aggravation of tinnitus, a known late effect among some young cancer survivors [82].

The choice for the secondary health-related outcome variables quality of life, self-esteem and self-efficacy in the present study was based on the results of a scoping review that mapped the concept, content and outcome of wilderness programs for childhood cancer survivors [41]. This scoping review described that wilderness programs seem to be most promising to contribute to the social involvement, self-esteem, self-confidence, self-efficacy, social support, and physical activity of childhood and AYA cancer survivors [41]. Participants in the present study were interviewed three months after study start with the aim to investigate their lived experiences and what the programs had meant for their health [28, 43]. Results of these qualitative analyses will be published elsewhere, but it is recommended that they are considered in the final selection of health-related outcome parameters in a future larger trail.

## Conclusions

In conclusion, the findings of this present pilot study demonstrate that it is feasible to conduct a RCT on a multi-component health promotion intervention such as the WAYA wilderness program. Both the wilderness and comparison holiday programs were acceptable for childhood and AYA cancer survivors. Serious adverse effects related to program participation were not reported and remaining effects were mild to moderate in severity and resolved within the study period. It was demonstrated that participation in the wilderness program was just as safe as spending vacation time at a Spa hotel. The findings of this study underscore the necessity to develop and apply other recruitment strategies in order to recruit sufficient participants for a future larger RCT. In the planning of such a large RCT it is recommended that the program content be adapted to stratify for the inclusion of cancer survivorship (childhood cancer versus AYA cancer), and to select health-related outcome variables based on the qualitative analysis of the lived experiences of participants in the program. This study provides critical and useful information for public health actors and outdoor/wilderness facilitators that plan to undertake wilderness expeditions with childhood and/or AYA cancer survivors. In addition, it informs researchers how to design a future larger RCT with the ultimate goal of investigating the effectiveness of wilderness programs on the health and well-being of childhood and AYA cancer survivors.

### Supplementary Information

**Additional file 1.** 

## Data Availability

Data related to this study will be made available upon request to the corresponding author.

## Abbreviations

AYA: Adolescent and Young Adult
AEs: Adverse Effects
AIC: Akaike Information Criterion
CI: Confidence Interval
CONSORT: Consolidated Standards of Reporting Trials
GSE: Generalized Self-Efficacy scale
MedDRA: Medical Dictionary for Regulatory Activities
MMQL: Minneapolis Manchester Quality of Life instrument
NRS: Nature Relatedness Scale
PT: Preferred Term
RCT: Randomized controlled trial
RR: Relative Risk
RSES: Rosenberg Self-Esteem Scale
SOC: System Organ Class
WAYA: Wilderness program for childhood, Adolescent and Young Adult cancer survivors
