# Supplementary material for: A randomized controlled pilot study assessing feasibility and safety of a wilderness program for childhood, adolescent, and young adult cancer survivors: the WAYA study
Source: BMC Public Health. 2023 Aug 8;23:1504. doi: 10.1186/s12889-023-16408-x (PMC10410899; doi:10.1186/s12889-023-16408-x)
Supplement: Supplementary file 1 — Additional file 1. [file 12889_2023_16408_MOESM1_ESM.pdf]

AE - Wilderness

Preferred term	Frequency	Percent
Tiredness	22	19,5
Insect bite	9	8
Situational anxiety	9	8
Constipation	6	5,3
Tension Headache	6	5,3
Back pain	5	4,4
Neuropatic pain	5	4,4
Bruise	4	3,5
Blister	4	3,5
Feeling anxious	3	2,7
Insomnia	3	2,7
Muscle soreness	3	2,7
Sunburn	3	2,7
Anxiety	2	1,8
Cut wound	2	1,8
General body pain	2	1,8
Headache	2	1,8
Knee pain	2	1,8
Mild neurocognitive disorder	2	1,8
Pain in hip	2	1,8
Shoulder pain	2	1,8
Stomachache	2	1,8
Abdominal pain	1	0,9
Abdominal pain aggravated	1	0,9
Buttock injury	1	0,9
Dizzines	1	0,9
Exhaustion	1	0,9
Hand injury	1	0,9
Mental exhaustion	1	0,9
Migraine	1	0,9
Scratch	1	0,9
Sleeplesness	1	0,9
Stiff joint	1	0,9
Stomach cramps	1	0,9
Wrist pain	1	0,9
	113	100

AE - Wilderness

System Organ Class	Frequency	Percent
General disorders and administration site conditions	25	22,1
Psychiatric disorders	25	22,1
Injury, poisoning and procedural complications	24	21,2
Musculoskeletal and connective tissue disorders	16	14,2
Gastrointestinal disorders	11	9,7
Nervous system disorders	11	9,7
Skin and subcutaneous tissue disorders	1	0,9
	113	100

AE - Holiday

Preferred term	Frequency	Percent
Tiredness	56	39,7
Headache	19	13,5
Sleeplesness	9	6,4
Insomnia	8	5,7
Mild neurocognitive disorder	8	5,7
Situational anxiety	7	5
Depressed mood	3	2,1
Tinnitus	3	2,1
Ankle sprain	2	1,4
Back pain	2	1,4
Feeling anxious	2	1,4
General body pain	2	1,4
Muscle soreness	2	1,4
Stomach ache	2	1,4
Stress	2	1,4
Sunburn	2	1,4
Allergic eczema	1	0,7
Anxiety reaction	1	0,7
Dizzines	1	0,7
Eczema aggravated	1	0,7
Knee pain	1	0,7
Localised pain	1	0,7
Neck pain	1	0,7
Non-cardiac chest pain	1	0,7
Panic attack	1	0,7
Restlessness	1	0,7
Skin abrasion	1	0,7
Urinary tract infection	1	0,7
	141	99,8

AE - Holiday

System Organ Class	Frequency	Percent
General disorders and administration site conditions	59	41,8
Psychiatric disorders	33	23,4
Nervous system disorders	29	20,6
Musculoskeletal and connective tissue disorders	9	6,4
Ear and labyrinth disorders	3	2,1
Injury, poisoning and procedural complications	3	2,1
Gastrointestinal disorders	2	1,4
Skin and subcutaneous tissue disorders	2	1,4
Infections and infestations	1	0,7
	141	99,9
